# A Study on the Data Compression Technology-Based Intelligent Data Acquisition (IDAQ) System for Structural Health Monitoring of Civil Structures

**DOI:** 10.3390/s17071620

**Published:** 2017-07-12

**Authors:** Gwanghee Heo, Joonryong Jeon

**Affiliations:** Department of Civil and Environment Engineering, Konyang University, 121 Daehak-ro, Nonsan, Chungnam 320-711, Korea; heo@konyang.ac.kr

**Keywords:** data compression technology, embedded software technology, artificial filter bank, band-pass filter optimizing algorithm, peak-picking algorithm, reconstruction error, compressive ratio, spectrum error, structural health monitoring

## Abstract

In this paper, a data compression technology-based intelligent data acquisition (IDAQ) system was developed for structural health monitoring of civil structures, and its validity was tested using random signals (El-Centro seismic waveform). The IDAQ system was structured to include a high-performance CPU with large dynamic memory for multi-input and output in a radio frequency (RF) manner. In addition, the embedded software technology (EST) has been applied to it to implement diverse logics needed in the process of acquiring, processing and transmitting data. In order to utilize IDAQ system for the structural health monitoring of civil structures, this study developed an artificial filter bank by which structural dynamic responses (acceleration) were efficiently acquired, and also optimized it on the random El-Centro seismic waveform. All techniques developed in this study have been embedded to our system. The data compression technology-based IDAQ system was proven valid in acquiring valid signals in a compressed size.

## 1. Introduction

In general, infrastructure is always exposed to unexpected weather conditions and consequent possibility of damage and strain as well as natural aging, and so its life span decreases [1,2]. As the size of structures is recently becoming larger, casualties and property damage could be accordingly larger in the event of an accident. In order to be well prepared for such threats and secure structural safety, there have been many studies on structural health monitoring (SHM) which makes it possible to inspect structural conditions on a regular basis and detect damage early for an effective preparation against an unexpected disaster or situation [3,4,5,6,7,8]. On a long-term basis, SHM technology can reduce the time and efforts required for the maintenance of structures [9]. For these reasons, it has recently been introduced to the construction of large bridges, skyscrapers and other major infrastructure. Examples of the application of the SHM technology to bridge construction include the Great Belt East Bridge [10] in Denmark, the Bill Emerson Memorial Bridge [11] in the U.S. and the Hakucho Bridge [12] in Japan. Examples of the application of the technology to skyscrapers are a 17-story building at the UCLA in the U.S. [13] and Republic Plaza [14] in Singapore.

In order to inspect structural conditions and early detect any damage using SHM technology, a decent SHM system must be developed. According to previous studies, most of SHM systems use wired sensor and measuring systems to secure a vast number of structural response data from sensors of various types [15] The wired SHM system, using cables for transmitting structural responses from sensors, is advantageous in acquiring data in a stable manner. Since data loggers are expensive, however, the system requires a large amount of initial cost for its installation. In addition, since power equipment and sensor location are mostly determined at the stage of design and construction, it is not easy to adjust or change the system later. In the case when new sensors need to be installed or old ones relocated, they all must be re-cabled, making the wiring complicated. If sensors are installed at a distance, then noise should also be considered. To overcome these practical and economic limitations of conventional wired SHM systems, Spencer [16] and Lynch [17] proposed a wireless SHM system based on a wireless sensor network. For example, Nagayama [18], Rice et al. [19] and Park [20] developed the wireless sensor node ‘Imote2’ through ANCRiSST and applied it for SHM and assessment of the structural state of a real bridge. In addition, Kurata et al. [21] developed ‘Narada’ using solar panels and sub-networks and applied it to New Carquinez Bridge in the U.S. for the purpose of SHM. Those examples proved that the wireless SHM system was effective in acquiring structural responses from multiple points on a large structure using wireless sensor nodes and wireless networks. Despite its various obvious advantages, conventional wireless SHM systems are not easy to restructure for either extension or simple change because they are designed and developed with limited resources to carry out only the sensor node-targeted functions. As each sensor node has an independent controller, it also requires a lot of time and efforts if a measuring logic is updated (micro-programmed). Its capacity could be additionally limited in processing consecutive measurement data on a real time basis. To make SHM efficient, therefore, there should be a user-friendly, high-performance SHM system which can: (i) acquire and process a large amount of diverse data on a real-time basis; (ii) easily edit measuring logic quickly and accurately and expand its functions and (iii) provide system safety and data quality up to the level of the commonly used (wired) measuring system even with the proven RF method. For these purposes, a software design technology-adopted, embedded software technology (EST)-based data acquisition (DAQ) system could be an alternative, overcoming the limitations of the conventional hardware-based wired and wireless measuring systems.

The EST-based DAQ system can adopt diverse RF systems in a flexible manner and easily expand multi-input/output channel (I/O) for multiple kinds and amounts of measurement. With the memory as large as a high-performance personal computer and with a high-performance CPU included, it is effective in acquiring, computing, processing and storing large data on a real-time basis. If necessary, it can be converted into a standalone system and operated as a sub-SHM system. Thanks to the adoption of the EST, in particular, the system can design and develop diverse logics (e.g., function, algorithm, etc.) which are needed in the acquisition, process and transmission of structural response signals and embed it within the DAQ system. Furthermore, the embedded logic can be operated under the real-time operating systems (RTOS). After all, this kind of EST-based DAQ system can effectively improve the weakness of the conventional hardware-based wired and wireless SHM system and considerably reduce time and efforts needed to develop a user-centered, high-performance SHM system which is directly applicable to actual structure.

Meanwhile, even though the EST-based DAQ system is capable of acquiring and storing a large amount of data, bottlenecks, data losses, or data delays can occur because RF protocols (e.g., Bluetooth, Zigbee, Wi-Fi, etc.) are technologically limited in receiving and transmitting data. The RF for a real-time SHM is supposed to work well under long distance, high speed and multi-channel conditions, but it is not so advanced yet. To develop a SHM system by overcoming these RF limitations, Peckens et al. [22] proposed a data compressing technology, a hardware filter bank imitating the human hearing mechanism. However, the hardware filter bank designed and developed for data compression for one particular infrastructure would not be valid anymore when the SHM system is applied to other structures, so a new hardware filter bank would have to be designed and fabricated [23,24]. When the bandwidth of dynamic responses (acceleration) changes in another SHM target structure, the SHM system with this kind of hardware filter bank would become inefficient from a practical standpoint. Such a weakness can be effectively improved through a design and development of a software type of filter bank which can edit/change its logic in a flexible manner. Especially, a digital type of software filter bank can be embedded into the DAQ system by coding the conventional hardware filter bank’s logic into high-level language, and thus it is basically different from the an analogy type of H/W filter bank in the wireless SHM in terms of filter band design method.

In this study, a software type of filter bank named ‘artificial filter bank (AFB)’ was developed for data compression. The AFB can selectively compress only the acceleration signals. Then, the developed AFB was completely embedded into the EST-based DAQ system, and an RF-based SHM system was developed and named an ‘intelligent data acquisition (IDAQ)’ system. To assess its validity, finally tests were conducted based on a random signal (the El-Centro seismic waveform). From the tests, it was found that the IDAQ system having AFB was valid in acquiring dynamic responses (only acceleration) for the SHM in a compressed size, successfully substituting the conventional hardware-based wired and wireless SHM system. In addition, it was confirmed that the system would be available as a user-centered SHM system because the measuring logic requested in a software design system can be easily and quickly developed and embedded.

## 2. The IDAQ System for Structural Health Monitoring

**Figure 1:**
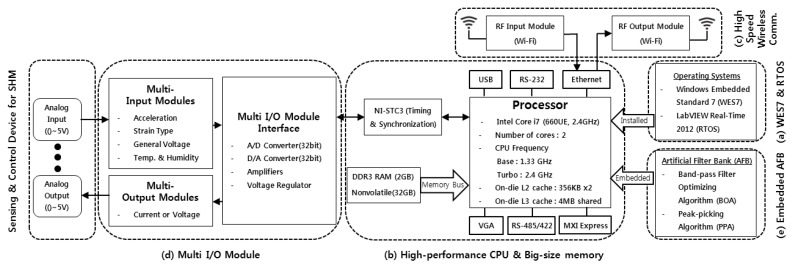
Design of the IDAQ system.

For efficient SHM on structures, this study develops an IDAQ system which is distinguished from a conventional hardware-based wired and wireless SHM system in the following features and advantages:

First, this study adopts the leading EST ‘Windows Embedded Standard 7 (WES7)’ and the TROS ‘LabVIEW Real-Time 2012 (LabRT2012)’ as shown in Figure 1a. The adoption of WES7 makes it possible to code the requested logics and functions in high-level language and embed them into the DAQs system. With the adoption of LabRT2012, the DAQ system can likewise be operated in a standalone manner. Then, the targeted structural response can be acquired and processed on a real-time basis, using the diverse logics and functions coded in the LabVIEW. After all, the introduction of EST and RTOS can innovatively reduce time and efforts needed to establish and operate the system and to develop and implement the measuring logics and functions needed for efficient SHM. 

Second, this study employs a high-performing CPU such as 32 GB memory, 2 GB RAM and Intel core i7 as shown in Figure 1b. Thanks to the introduction of this kind of high-performing PC-level controller, a user is able to efficiently save, compute and process large measurement datasets for SHM. After all, optimum measuring conditions can be provided for efficient SHM. 

Third, this study adopts a Wi-Fi communication module which enables high-speed mobile communication as shown in Figure 1c. The introduction of Wi-Fi-based access points (APs) makes it possible to transmit large data at high speed and expand to multiple channels. If necessary, a wireless network could be configured for high-speed mobile communication even at a distance by adding low cost APs. In addition, practicality-considered RF communication functions are secured to enable closed-operation (e.g., feedback vibration control, etc.) based on structural response according to users’ purpose and demand through two-way communication. Mobile communication-aimed AP is also configured as an independent hardware from the IDAQ system so that the AP of other wireless communication systems (e.g., Zigbee, Bluetooth, etc.) can be easily changed or adopted depending on user demand. 

Fourth, this study takes in a Multi-I/O by which it can expand channels according to a type of sensor, as shown in Figure 1d. With the introduction of Multi-I/O, various data needed for SHM can be measured and operated in an integrated manner using the IDAQ system. In particular, it fundamentally solves the problems of conventional SHM—high initial cost for system development and data synchronization. 

Lastly, this study adopts a data compressing technology of dynamic responses, ‘AFB’, as illustrated in Figure 1e. Here, the AFB is developed to acquire effective dynamic responses in a compressed size and wholly embedded into the IDAQ system. The introduction of this kind of AFB helps the IDAQ system effectively reduce a large volume of dynamic responses needed for SHM. In addition, more efficient data transmission is expected than in the RF system. Therefore, this system can be a great alternative for the RF-based real-time SHM. As stated above, the IDAQ system is compared to the conventional wired and wireless SHM system. Its structure can be represented in a diagram as shown below:

## 3. An Artificial Filter Bank (AFB) for the IDAQ System 

In this study, the IDAQ system is developed for efficiency. As described in Section 2 above, the IDAQ system features a CPU and memory capacity as high as the latest high-performance personal computer allows. It also has the following: WES7 based on EST, LabRT2012 based on RTOS, multi I/O for diverse/multiple measurement in an integrated manner, Wi-Fi AP for high-speed mobile communication, AFB (data compression technology of dynamic responses (only acceleration)). In this section, the mechanism of AFB developed for the IDAQ system is described. The AFB can selectively compress only the acceleration signal. AFB’s specific technologies band-pass filter optimizing algorithm (BOA) and peak-picking algorithm (PPA) are also briefly explained. The index such as reconstruction error (RE), compressive ratio (CR) and spectrum error (SE) by which the AFB’s performance is assessed are also described.

### 3.1. Artificial Filter Bank (AFB) Mechanism 

**Figure 2:**
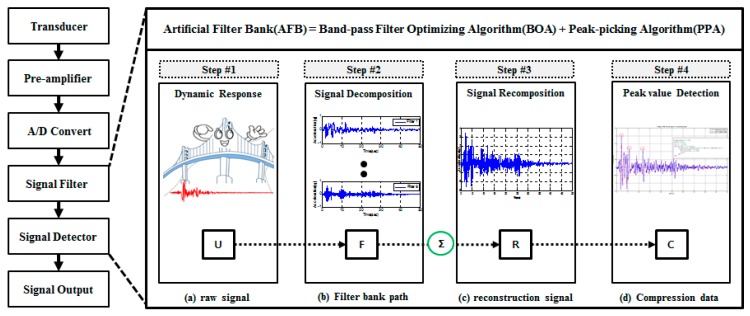
Mechanism of AFB for IDAQ system : (**a**) raw signal; (**b**) filter bank path; (**c**) reconstruction signal; (**d**) compression data

In the field of signal processing, a filter bank is defined as a special filtering arrangement that filters certain frequency elements (information) of interest only based on input signal standards. It handles a series of processes such as classifying input signals by frequency, reconfiguring and printing them. That is, it arranges input signals into each appropriate area of frequency and outputs the rearranged ones. The process of rearrangement means a ‘decomposition of signals’ by decision of each filter, and that of output becomes a ‘synthesis of signals’ occurring during filtering. Such a filter bank can be differently designed depending on purposes and frequency levels. As the number of band-pass filters in the filter tank increases and they become denser, filtering signals get more accurate than the input signal. However, data operating and processing efficiency declines when the number of band-pass filters increases. Therefore, there is a need to optimize the filter bank. To optimize a filter bank, primary design factors such as number of band-pass filters, bandwidth and spacing should be considered. Here, the optimization of filter bank is to determine the three design factors to build the best reproduction capability based on the targeted input signal. For this, numerical and repetitive operations are required under different combinations of the three design factors. Meanwhile, the signals (Figure 2b) decomposed by passing through the band-pass filter in the filter bank and also its synthesized and reconstructed ones (Figure 2c) include only certain frequency information shown as in Figure 2a. Therefore, even though the filter bank can be valid in data acquisition, it will have only the same size of data with the same sampling spacing as the input signals because of the specific features particular to band-pass filters. In other words, although certain reconstructed signals may be selectively better acquired than input signals, the size of the acquired data remains the same. After all, it turns out to be not so efficient in terms of RF communication-based transmission and management of acquired data.

Therefore, to efficiently acquire the dynamic response of structure using the limited capacity of RF communication, data compressing technology is required. What is of most concern in developing a data compression technology for dynamic responses is to reflect frequency information just like the filter bank above. Based on several design factors and considerations, this study developed an AFB by combining the BOA aimed to optimize the filter bank with the PPA designed to compress the reconstructed signals obtained from the filter bank. The mechanism of the AFB developed for the IDAQ system is shown in Figure 2 below:

### 3.2. Band-Pass Filter Optimization Algoritnm for AFB

The reconstructed signals in Figure 2c can be expressed in a form of combining individual filtering signals which the filter bank passes, as stated in Equation (1) below. Then, the filter bank selects some data of certain frequency among the original (raw) signals of diverse frequency, and classifies them into several parts for filtering and output. Here, in order that the reconstructed signals sufficiently reflect initial input signals, it is required to design the filter bank in an optimum manner. With a goal of optimizing the filter bank, this study develops a BOA to determine three design conditions (number of band-pass filters, bandwidth and spacing) in the band-pass filter.

The BOA’s calculation process can be divided into the following four steps: First, reconstruction errors are comparatively numbered with the assumed number of band-pass filters based on the initial input signal. Second, the number of band-pass filters is set as the optimum condition at the point where the slope of the reconstruction error is inflected. Third, a reconstruction error is calculated by changing the bandwidth and the central frequency interval, on the basis of the number of band-pass filters. Fourth, optimal is the bandwidth and mean frequency interval which have the minimum reconstruction error.

**Figure 3:**
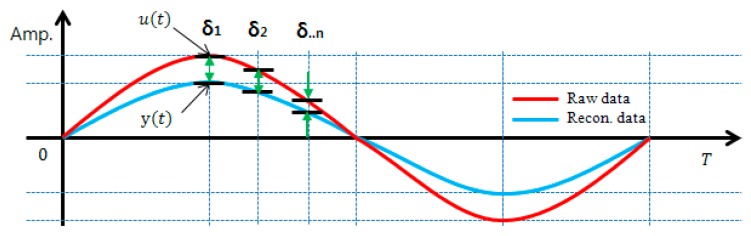
Concept of reconstruction error (RE).

In this study, the RE, which is required for BOA’s calculation process to optimize the filter bank, is defined as shown in Equation (2) from the concept in Figure 3 below. The RE defined in Equation (2) is represented by the relative difference of absolute values between original and reconstructed signals. As the RE is close to ‘0’, it means the original signal is successfully simulated and further, the filter bank is judged to have excellent reconstruction ability:

(1) $$u\left(t\right)\approx y\left(t\right)=\overset{N}{\underset{i=1}{\sum }}y_{i}\left(t\right)$$

(2) $$\mathrm{RE}=\;\frac{\int_{0}^{T}\left|u\left(t\right)-y\left(t\right)\right|/\left|u\left(t\right)\right|}{T}=\;\frac{\int_{0}^{T}\left|\delta_{i}\right|/\left|u\left(t\right)\right|}{T}$$

where, u(t) is an original signal by response time while y(t) and *T* represent a reconstructed signal by response time and a total length (s) by response time, respectively. Even though Peckens and Lynch [22] used $l_{2}-norm$ for RE, it is a scale for the comparison of the RE’s total energy against the original signal. To clearly express the reconstruction effect of the time response of the reconstructed signal including peak values, this study states the reconstruction signal’s error against original signals on absolute scale.

### 3.3. Peak-Picking Algorithm Based on the Central Difference Method

As mentioned above, to efficiently acquire the dynamic responses of structure using RF communication technology with limited performance, data compression technology is required besides selective sorting of data. For this, this study develops a PPA to reduce the size of the acquired data while including information of certain frequencies of interest pertaining to the characteristics of the dynamic responses. The developed PPA derives peak values from reconstructed signals, not from original signals. Then, the calculation process for data compression can be dramatically reduced by deriving peak values based on the reconstructed signal, a set of filtering signals, not the peak value of the filtered signal.

The PPA’s calculation process can be summarized as follows: first, calculated reconstructed signal is read from the optimized filter bank. Second, the reconstructed signal is classified into three (3) data groups. Third, each group’s derived function is calculated based on three data in each group. Fourth, peak values (time and measuring data) are derived by assessing the slope (sign) of each group’s derived function.

**Figure 4:**
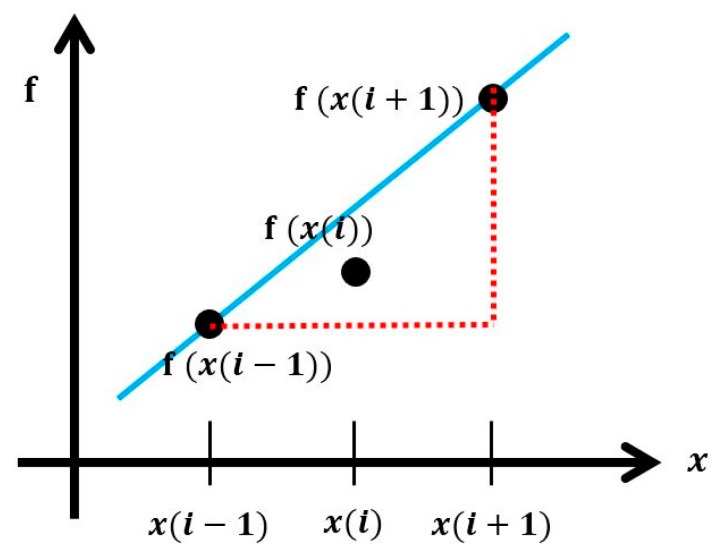
Concept of central difference method.

In this study, a central difference method is used as a way to extract peak values while PPA is calculating. Here, the difference method is classified into backward difference, central difference and forward difference under the assumption that the relation among neighboring data is linear. If the neighboring data is spaced suitably enough to meet the assumption, a derived function with the lowest error can be calculated using the central difference method. Figure 4 above reveals the central difference method’s conceptual diagram, and a derived function can be calculated through Equation (3):

(3) $$f^{\prime }\left(x\left(i\right)\right)=\;\frac{f\left(x\left(i+1\right)\right)-f\left(x\left(i-1\right)\right)}{x\left(i+1\right)-x\left(i-1\right)}$$

Based on the size of the reconstructed data determined through the Equation (1) above, the effects of data compression through PPA can be defined by comparing a relative data size with the extracted peak value. For this, this study defines data CR as stated in Equation (4) below:

(4) $$\mathrm{CR}=\;\frac{NS_{0}-\;NS_{C}}{NS_{0}}$$

where, $NS_{C}$ is the number of compressive signal data while $NS_{0}$ refers to the number of reconstructed signal data. If the CR gets closer to ‘0’, the compression effect increases. Here, the defined CR is used for the assessment of the compression effects against reconstructed signal.

## 4. Artificial Filter Bank (AFB) Optimization 

The previous chapter defined AFB as a data compressing technology of dynamic responses to acquire the efficient dynamic responses (only acceleration) of structures using the IDAQ system by combing both BOA and PPA. In this chapter, the AFB is optimized based on a random signal (the El-Centro seismic waveform) which is commonly used in the construction industry to assess the IDAQ system’s applicability for SHM on large architectural structures.

### 4.1. Reference Signal for AFB Optimization 

**Figure 5:**
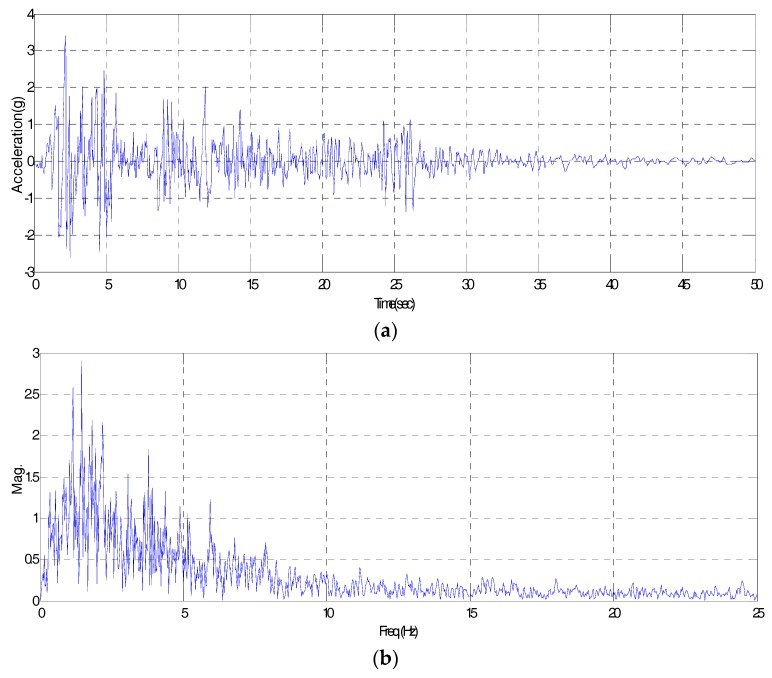
El-Centro seismic waveform for reference signal of AFB: (**a**) Time domain; (**b**) Frequency domain.

It is well known that El-Centro, Kobe and Northridge are the leading random seismic waveforms used in the construction industry. They represent an unexpected circumstance which may be occur when designing, constructing and managing structures. To assess the applicability of the IDAQ system for SHM into large structures, therefore, the ‘El-Centro’ seismic waveform was chosen as an original signal for the optimization of AFB among the random seismic waveforms mentioned above. Figure 5 below shows the El-Centro seismic waveform (SAC Name: LA02 (1940, SE)) used as an original signal, which is expressed in time and frequency responses. According to Figure 5a, a seismic situation lasts for a short period of time (approximately one minute in the case of the El-Centro event). In Figure 5b, diverse frequency factors are distributed across the frequency bands. In particular, the frequency of interest is concentrated in less than 10 Hz. Here, if the seismic situation in Figure 5 is assumed, the dynamic responses of the target structure are measured as a type which includes the target structure’s own frequency factors in the conventional seismic waveform’s random frequency factors. 

For SHM on structures, after all, the target structure’s original frequency factors in the random frequency factors should be fully discovered. Because large structures in the construction industry usually have relatively flexible behavioral characteristics, the range of the target mode needed for SHM can be limited to a certain range of frequency (e.g., below 10 Hz). From this perspective, the El-Centro seismic waveform selected as an original signal could be used as a reference response which can wholly express the large structure’s rage of frequency concerned (below 10 Hz). Under the assumption that an El-Centro seismic situation has these frequency characteristics, this study designs the AFB in an optimum manner based on the El-Centro seismic waveform (total time length: 50 s, delta T: 0.02 s, total frame length: 2500).

### 4.2. Optimizing Desing of AFB Using a BOA

**Figure 6:**
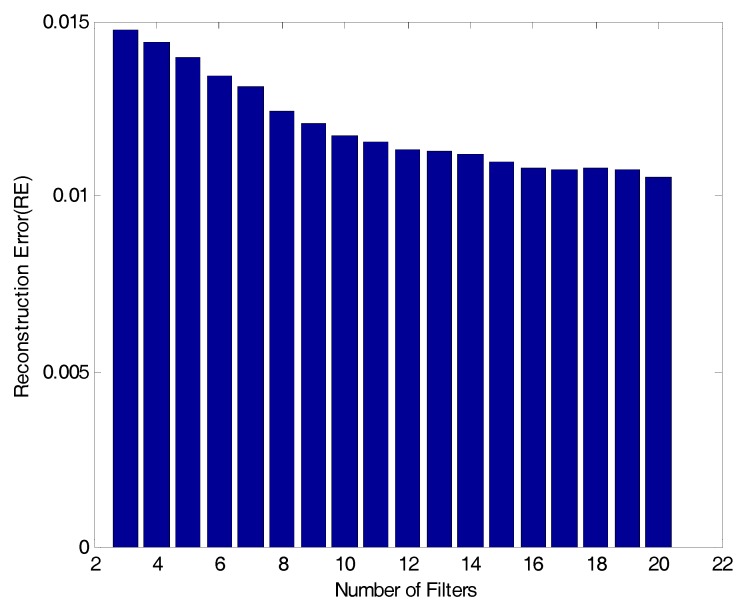
RE results to number of filters.

To derive the optimum conditions for a filter bank, this study attempts to determine three design factors (number of filters, bandwidth and spacing) for the band-pass filter which would be used in the filter bank. First, to determine the number of band-pass filters, a RE was calculated using Equation (2) by changing the number of filters from 3 to 20 (18 cases in total) through the BOA. Figure 6 below presents the RE results by the number of band-pass filters for the 18 cases. According to the figure, when the number of band-pass filters is less than 10, the RE largely depends on the filter conditions. When it is 10 or more, on the contrary, the RE is small depending on the filter conditions. In addition, the fluctuation in RE is large when the number of band-pass filters ranges from 3 to 10. When it is 10 or more, in contrast, the fluctuation is relatively small. To design an efficient filter bank, after all, it is advantageous to reduce a RE by decreasing the low number of band-pass filters. Therefore, the number of band-pass filter is set to ‘10’ in this study. 

**Figure 7:**
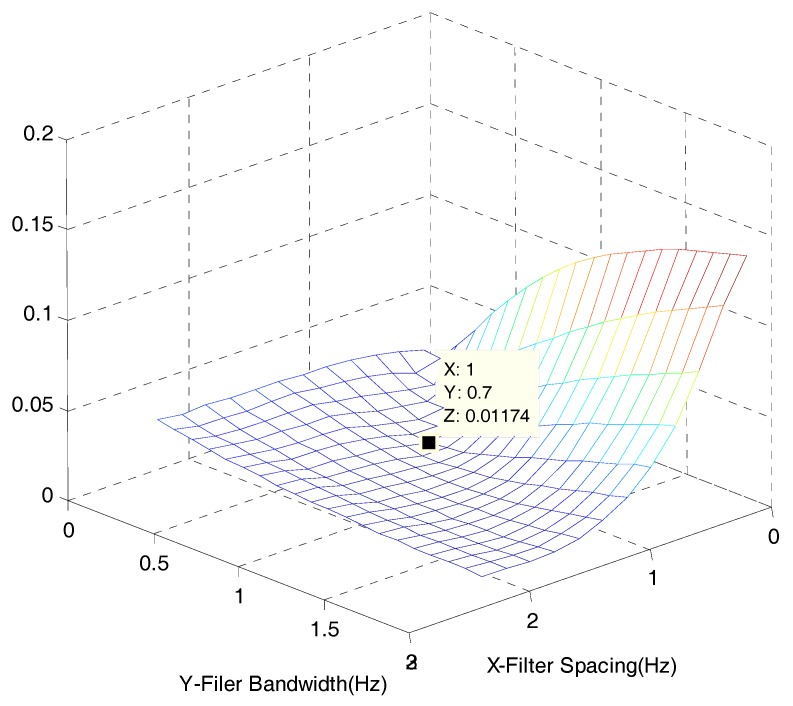
RE results for 10 filters.

Then, to determine bandwidth and spacing, a RE by the bandwidth and spacing is calculated using the 10 band-pass filters. The bandwidth for the band-pass filter is divided into 20 categories (0.1 to 2.0 Hz, increase by 0.1 Hz). In addition, spacing for the band-pass filter is classified into 12 conditions (0.2 to 2.4 Hz, an increase by 0.2 Hz). Based on the bandwidth and spacing conditions, REs on a total of 240 cases are iteratively calculated, and the results are stated in Table 1 below. In addition, they are illustrated in Figure 7. According to Table 1 and Figure 7, when 10 band-pass filters are used, the bandwidth for the band-pass filter with a minimum RE is 0.7 Hz. Then, the spacing for the band-pass filter is 1.0 Hz. In conclusion, the conditions of the band-pass filter in the filter bank for the optimum acquisition of El-Centro seismic waveforms in Figure 5 are determined as follows: 10 filters, 0.7 Hz in bandwidth and 1.0 Hz of spacing.

**Figure 8:**
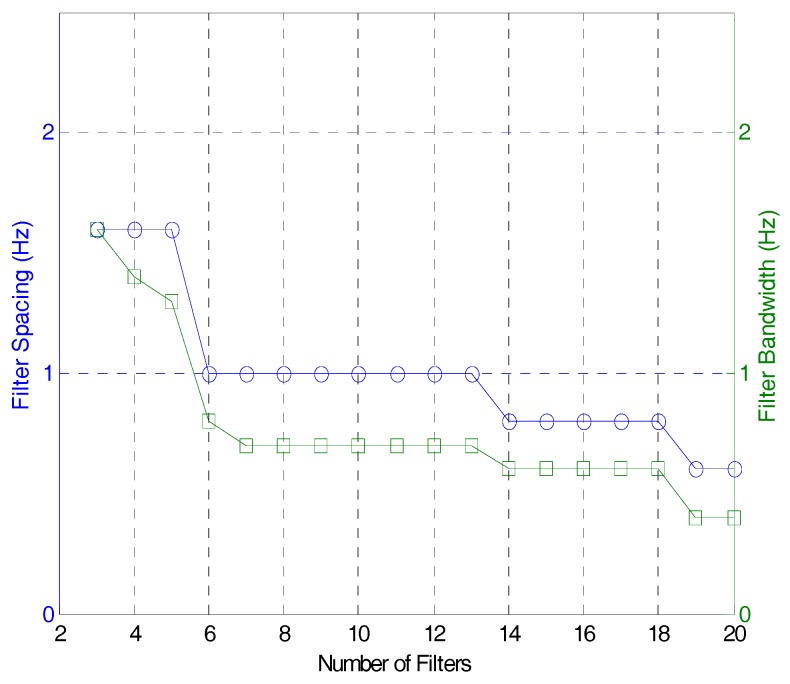
Bandwidth and filter spacing for different numbers of filters.

Meanwhile, Figure 8 reveals a change in the bandwidth and spacing of the band-pass filter as a consequence of the change in the number of band-pass filters. According to Figure 8, as the number of filters increases, both bandwidth and spacing decrease. On the other hand, bandwidth and spacing increase when the number of filters decreases. To get a maximum amount of information from the response using a limited number of band-pass filters, then, the filter bank should be optimized. To determine the number of band-pass filters, and also bandwidth and spacing at the same time, iterative calculation is required.

### 4.3. Data Compression Using a PPA

In Section 3.2, the optimum conditions of a filter bank (number of band-pass filters, bandwidth and spacing) are derived based on the BOA. The optimized filter bank produces reconstructed signals which can simulate original signals most closely. If a filter bank is optimized using a BOA, after all, the most accurate reconstructed signal is expected, even for random original signals. Meanwhile, even though the reconstructed signal which the optimized filter bank passes has the minimum RE with original signal, the reconstructed signal’s size is the same as that of the original signal in terms of sampling rate. For SHM on large structures, in particular, there might be a high demand for a multi-channel-based precision measurement with a high sampling rate. Because the large dynamic responses acquired on a real-time basis is transmitted using the RF with limited performances, it can cause a data loss because of bottleneck. To get large dynamic responses based on the RF, a data compressing technology is essential.

**Figure 9:**
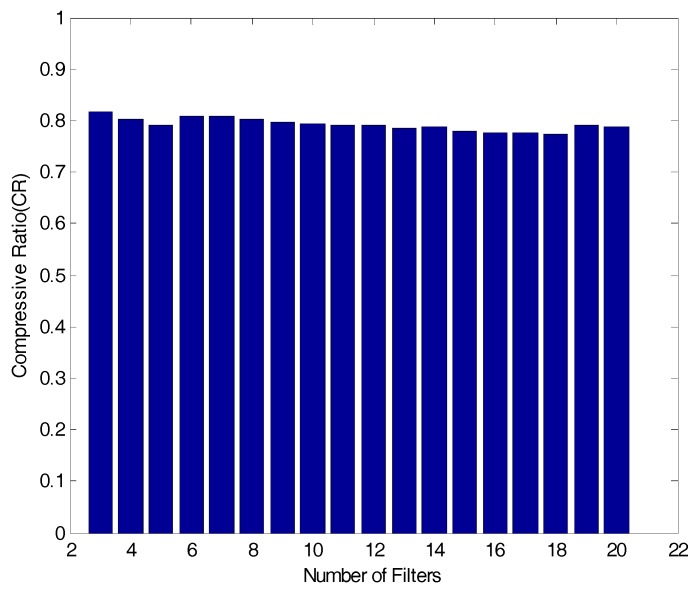
CR results for number of filters.

For data compression, this study develops a PPA, using a central difference method mentioned in Figure 3 and Equation (3). For a reference signal for the detection of the peak value, a reconstructed signal which the filter bank passes is used. Then, the reconstruction sign’s peak values are determined through changes in negative and positive signs on the derived function of the reconstruction signals calculated in sequence. Figure 9 below reveals CR results by the bandwidth and spacing in which the RE reaches the lowest level when the number of band-pass filters in Figure 6 changes:

According to the Figure 9 above, CR is 0.8176 when the number of band-pass filters is 3. The ratio drops to 0.788 when the number of band-pass filters increases to 20. Here, fluctuations in the CR over changes in the number of band-pass filters are minor. In particular, when the number of bass-pass filters is 10 (the optimum condition for the filter bank), the CR is 79.44, anticipating about 80% of data compressing effects.

### 4.4. Development of AFB

**Figure 10:**
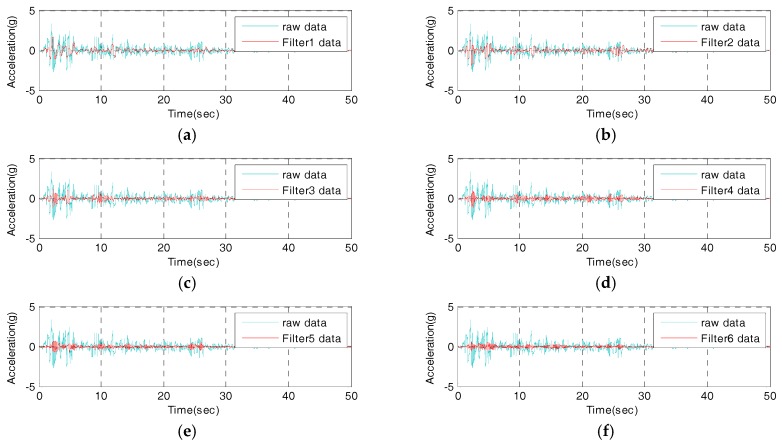

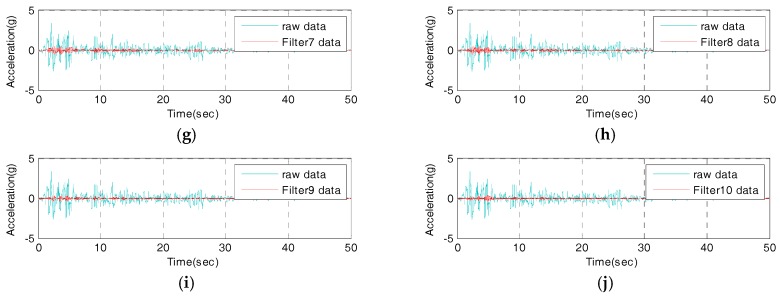
Time domain response of optimal condition AFB: (**a**) filter 1 data; (**b**) filter 2 data; (**c**) filter 3 data; (**d**) filter 4 data; (**e**) filter 5 data; (**f**) filter 6 data; (**g**) filter 7 data; (**h**) filter 8 data; (**i**) filter 9 data; (**j**) filter 10 data.

**Figure 11:**
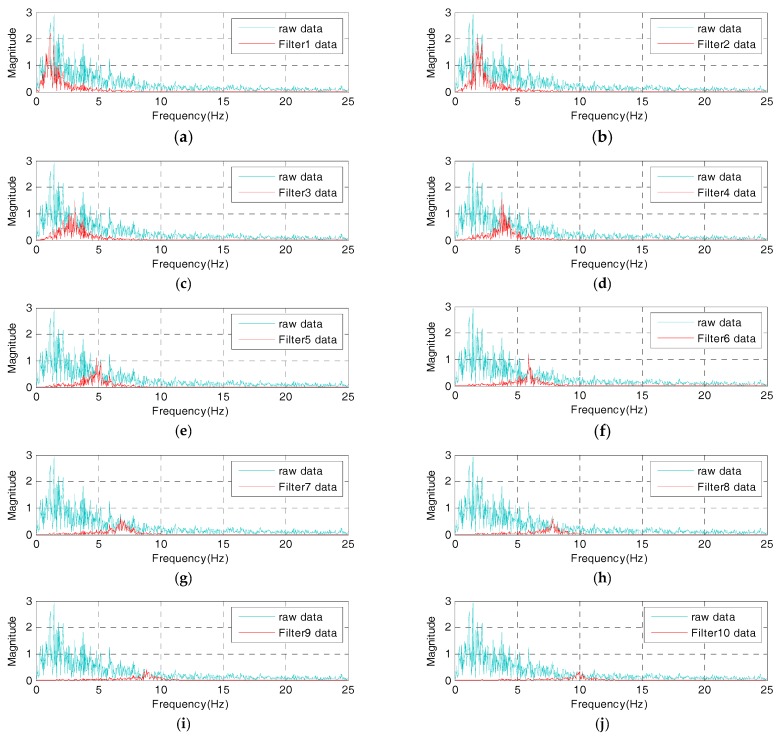
Frequency domain responses of optimal condition AFB: (**a**) filter 1 data; (**b**) filter 2 data; (**c**) filter 3 data; (**d**) filter 4 data; (**e**) filter 5 data; (**f**) filter 6 data; (**g**) filter 7 data; (**h**) filter 8 data; (**i**) filter 9 data; (**j**) filter 10 data.

In this study, prior to the IDAQ system’s test, an AFB logic which would be embedded into the IDAQ system is developed, and whether or not the AFB could be normally operated is examined. Then, the AFB can program band-pass filters using commercial programming language (e.g., C, C++, Matlab, JAVA, etc.). In this study, to develop an AFB, the Matlab M-code is adopted. In addition, the El-Centro seismic waveform is applied into the AFB. Then, filtering signals are estimated from each band-pass filter and compared to each other. Figure 10 and Figure 11 below are the time and frequency responses of the filter-bank of the determined optimum conditions where the number of band-pass filters is 10, bandwidth is 0.7 Hz, and spacing is 1.0 Hz. These two figures show that the filter bank is optimally designed using the determined conditions.

Here, it can be confirmed that the AFB is successfully designed under the determined optimum conditions (six band-pass filters, 0.6 Hz in bandwidth and 1.0 Hz of spacing). In addition, time and frequency information is more implicated in the filters 1 through 6. As a result, it is confirmed that the AFB-added IDAQ system optimized in the El-Centro seismic waveform is effective for the acquisition of dynamic responses and SHM on large and relatively flexible structures which have a range of frequency less than 6 Hz. If the AFB is designed with a random reference signal, not with the El-Centro seismic waveform, however, optimum conditions can differ. Then, a user can configure the AFB optimally by determining the reference signal for the purpose.

## 5. Estimation of the IDAQ System

**Figure 12:**
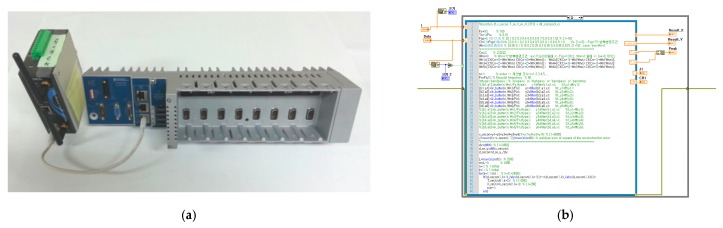
H/W and S/W for the IDAQ system: (**a**) EST-based DAQ (Controller); (**b**) Embedded AFB (optimal conditions).

In this chapter, the IDAQ system is completed by embedding the AFB developed under the optimum conditions into the EST-based DAQ system. Based on the El-Centro seismic waveform (SAC Name: LA02 (1940, SE)), then, the IDAQ system’s test is performed. As for the test, it is done through the IDAQ system’s input by forming the El-Centro seismic waveform using the LabVIEW’s function generator. Then, the AFB’s output signals (reconstructed signal, compressive signal, etc.) embedded in the IDAQ system was acquired wirelessly from the host PC. Finally, whether or not the IDAQ system properly carries out a series of targeted operations (real-time acquisition, decomposition, reconstruction, compression and transmission of dynamic responses (acceleration)) for SHM is assessed in a standalone manner. Figure 12a below reveals the logic of the AFB programmed based on the optimal conditions mentioned earlier, while Figure 12b shows the IDAQ system’s controller used for embedding the programmed AFB's.

In this study, NI’s cDAQ-9139 controller is used to completely embed (systematize) the developed AFB into the Matlab M-code. The cDAQ-9139 controller has a 1.33 (max: 2.4 GHz) dual core Intel i7 processor, 2 GB of RAM and 32 GB of data storage space in the stand-alone chassis to accomodate the IDAQ system mentioned in Section 2. Therefore, it offers a dramatically improved system environment for acquisition, processing and storage of high-speed and large data on a real-time basis. In addition, it features WES7 and Linux-based RTOS which guarantee the embedded system’s stability and real-timeness. Therefore, the developed AFB logic can be completely embedded and operated on a real-time basis. In addition, a MOXA SWK-3121 AP module designed for multi I/O is applied for an integrated multiple (various) measurement and high-speed and two-way RF (Wi-Fi) communication. Thus, the IDAQ system in this paper is designed to carry out a series of standalone operations for the real-time acquisition, decomposition, reconstruction, compression and transmission of large dynamic responses (acceleration) based on the built-in AFB.

### 5.1. Reconstructed Signal of the IDAQ System

**Figure 13:**
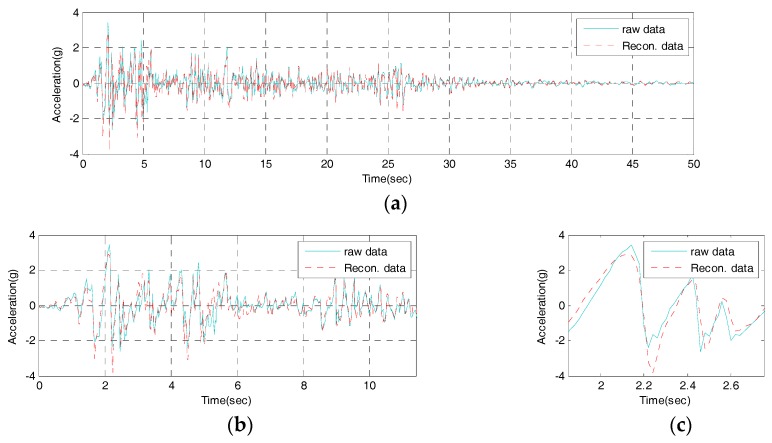
Time domain of the reconstructed signal from the IDAQ system: (**a**) Time from 0 to 50 s; (**b**) Detail 1; (**c**) Detail 2.

**Figure 14:**
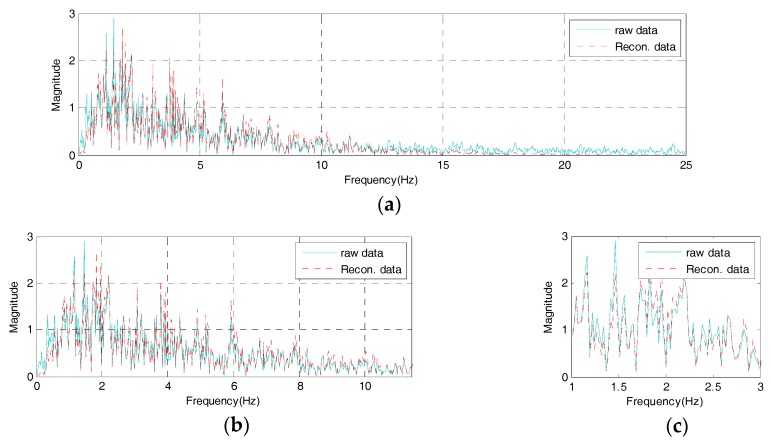
Frequency domain of the reconstructed signal from the IDAQ system: (**a**) frequency from 0 to 25 Hz; (**b**) Detail 1; (**c**) Detail 2.

To test the validity of the IDAQ system developed for the efficient SHM, a test is conducted. Then, the output signals, coming from original signals after inputting the El-Centro seismic waveform, are classified into reconstructed signals (a set of signals which have passed through the band-pass filter), and compressive signals which are made after abstracting only peak values based on reconstructed signals. Those two signals are assessed with RE in Equation (2) and CR in Equation (4) respectively. First, the time and frequency response of the reconstructed signals are estimated from the IDAQ system as shown in Figure 13 and Figure 14, respectively.

According to Figure 13, it is found that the time response of the reconstructed signals obtained under the optimum conditions sufficiently simulates that of the original signal, by means of only 10 band-pass filters. The estimated RE shows 0.01174, and approximately 70% of reconstruction capability is found against total original signals. In particular, the maximum acceleration is 3.417 g at 2.14 s, when the seismic acceleration of original signal is the highest. Then, the maximum acceleration of reconstructed signals is 2.878 g. Therefore, the reproduction capacity turns out to be 85% at the highest acceleration of the reconstructed signals, which is excellent.

**Figure 15:**
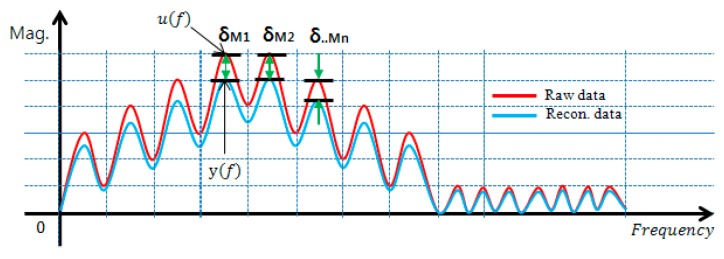
Concept of spectrum error (SE).

Meanwhile, the frequency response of the reconstructed signal which passed through the AFB embedded in the IDAQ system should reflect all range of optimized frequency information besides the reproduction capability on time response as shown in Figure 13. To assess the frequency response’s reproduction performances in the reconstructed signal, this study defines the reconstructed signals’ spectrum error (SE) as shown in Equation (5) from the concept in Figure 15.

(5) $$\mathrm{SE}=\;\frac{\int_{0}^{F}\left|u\left(f\right)-y\left(f\right)\right|/\left|u\left(f\right)\right|}{F}=\frac{\int_{0}^{F}\left|\delta_{Mi}\right|/\left|u\left(f\right)\right|}{F}$$

Here, u(f) is the original signal’s frequency response while y(f) refers to the reconstructed signal’s frequency response. In addition, ‘F’ represents the total length (Hz) of the spectrum estimated through the fast fourier transform (FFT) analysis. Then, if the SE in Equation (5) gets closer to ‘0’, the reconstruction effect on the reconstructed signal’s frequency response is excellent, just like the RE defined in Equation (2). The reconstructed signal’s SE from the frequency response of the reconstructed signal in Figure 14 is 0.01725. Compared to the total original signal’s frequency response, about 83% of spectrum reconstruction ability is found.

Also, the SE on the originally targeted frequency range (less than 10 Hz) is found to be 0.00928, showing about 91% of spectrum reconstruction capability, therefore the reconstructed signal estimated under the optimum conditions through the AFB can sufficiently simulate the original signal’s frequency response with 10 band-pass filters only. Specifically, it simulates all information on the original signal’s frequency range (less than 10 Hz). Based on the results above, the IDAQ system in this study works successfully with the AFB’s logic optimized in the El-Centro seismic waveform. It is also found to be effective in estimating reconstructed signals including mode information concerned which meets the AFB’s optimum conditions.

### 5.2. Data Compression of the IDAQ System

**Figure 16:**
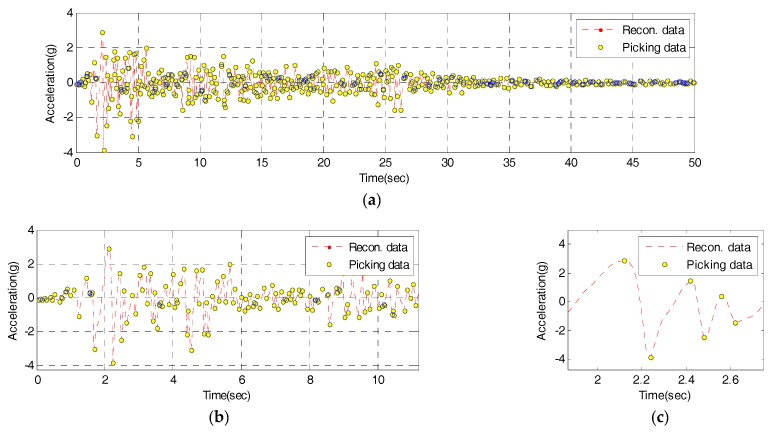
Peak-picking results of the reconstructed signal from the IDAQ system: (**a**) Time from 0 to 50 s; (**b**) Detail 1; (**c**) Detail 2.

To test the validity of the IDAQ system developed for the efficient SHM, then, the data compression ability was assessed. The reconstructed signal’s time response derived in Figure 13 is about 70% against the original signal while the reconstructed signal’s frequency response from Figure 14 is about 83% against the original signal in terms of reconstruction ability. Even so, the size of the estimated reconstructed signal’s time response is the same as the data size of original signal. To carry out SHM by acquiring a great number of dynamic response data (acceleration) using the limited RF, data compression technology is needed. For this, this study develops a PPA using the central difference method. Here, the PPA is designed to extract peak values only based on reconstructed signals. Figure 16 below reveals the peak-picking results of the reconstructed signal from the IDAQ system.

In Figure 16, the central difference method-based PPA is able to pick up only the reconstructed signal’s peak values. A total of 520 peak values are derived. The CR against 2500, the number of reconstructed signals for fifty seconds, is 0.7920. When PPA is combined with the AFB, about 80% of data are expected to be compressed under the conditions optimized with the El-Centro seismic waveform. Based on the results above, it is found that the IDAQ system operates well based on the AFB’s logic which is added to the PPA developed using the central difference method. Among the reconstructed signal’s time responses, then, only the peak values which represent frequency information (periodicity) are picked up so that it is proven valid in estimating compressed dynamic responses.

### 5.3. Compressive Signal of the IDAQ System

**Figure 17:**
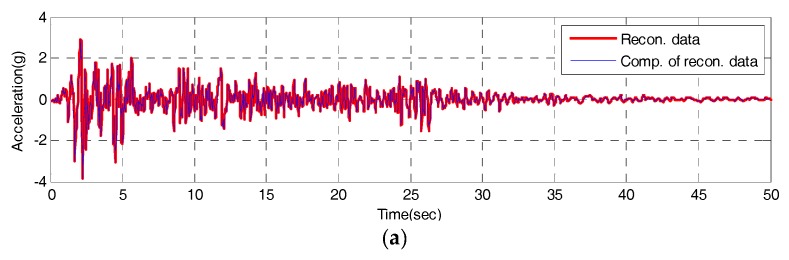

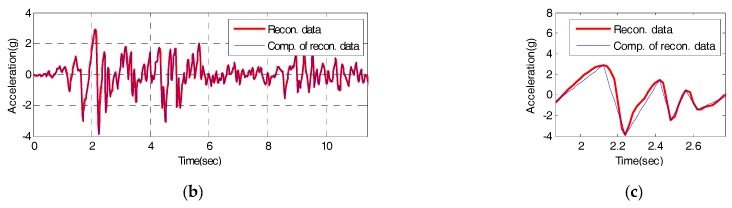
Time domain of compressive signal using peak-picking values: (**a**) Time from 0 to 50 s; (**b**) Detail 1; (**c**) Detail 2.

**Figure 18:**
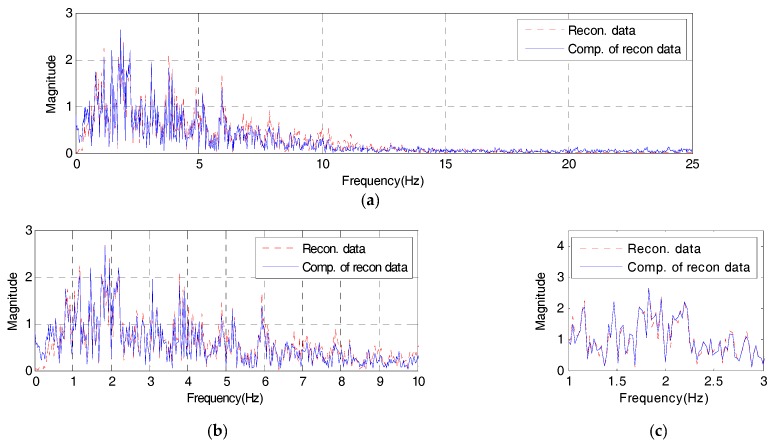
Frequency domain of compressive signal using peak-picking values: (**a**) frequency from 0 to 25 Hz; (**b**) Detail 1; (**c**) Detail 2.

To test the validity of the IDAQ system developed for the efficient, only the peak values (Figure 16) picked up from the IDAQ system are transmitted using the RF, and the test is performed to have them stored in the host PC. Then, the peak values stored in the host PC is to be restored into a signal-analyzable type for future SHM. In this study, the signals restored with peak values only are defined as ‘compressive signals’. For SHM, compressive signals of the same size (time length) as the spacing (delta t) equivalent to the original sampling rate are required. To crate compressive signals using the peaks only, which are acquired and stored based on irregular spacing, linear interpolation is used. This method can preserve peak values determined from the original PPA and create compressive signals which are the same in terms of reconstructed signal and spacing within the peak range. Figure 17 and Figure 18 below show the time and frequency domains of compressive signals restored using the linear interpolation, respectively.

In the compressive signal’s time response derived from Figure 17, the RE is 0.00756, compared to the reconstructed signal. As a result, the compressive signal is well able to restore data compared with the reconstructed signal (the restoration ability is approximately 81%). Specially, the reconstructed signal’s peak values picked up from the PPA are properly reflected and simulated. Nevertheless an RE occurs because a section of the empty time data without peak values is linearized in restoring the compressive signal’s time response using the linear interpolation. Because the peak values of time response are used as one of major interest information in executing SHM, the linear interpolation-based compressive signal restoration technology accomplishes the design goal.

**Figure 19:**
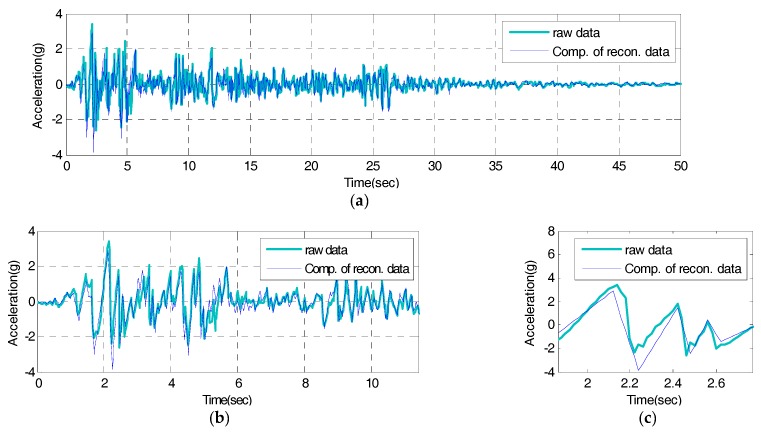
Time domain of raw signal vs. compressive signal: (**a**) Time from 0 to 50 s; (**b**) Detail 1; (**c**) Detail 2.

**Figure 20:**
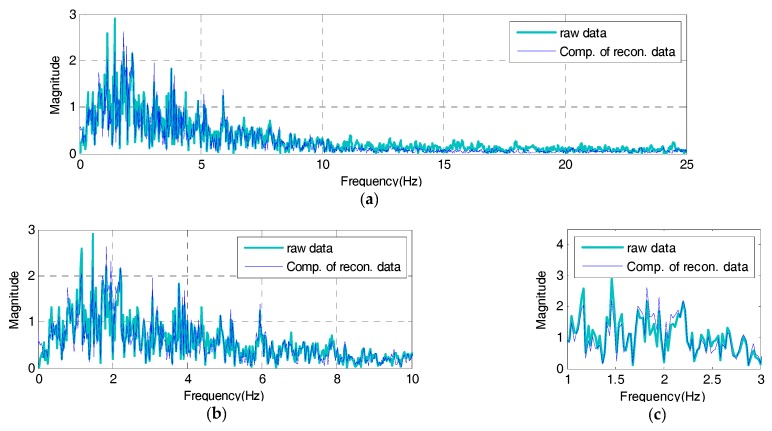
Frequency domain of raw signal vs. compressive signal: (**a**) frequency from 0 to 25 Hz; (**b**) Detail 1; (**c**) Detail 2.

While the reconstructed signal remarks especially mode information about frequency range of original signal (1 to 10 Hz) in Figure 14, the compressive signal expresses it all across the total frequency range of reconstructed signals as in Figure 18 because the peak values, the reference data of compressive signals, are derived throughout the reconstructed signal’s response time. The estimated compressive signal’s frequency response shows 0.01445 of SE, compared to the reconstructed signal. In comparison with the total original signal’s frequency response, about 85% of spectrum reconstruction ability is observed. Based on the results above, therefore, the compressive signal’s frequency response properly simulates all information throughout the reconstructed signal’s frequency range. Lastly, to test the validity of the IDAQ system developed for efficiency, the compressive signal’s time and frequency responses are shown in Figure 19 and Figure 20, respectively as the final results of the test based on the originally targeted original signals.

In Figure 19, the compressive signal’s time response generated based on the reconstructed signal’s peak values sufficiently simulates the original signal’s periodicity. The RE with the original signal is 0.01702, showing about 60% of reconstruction ability against the reconstructed signal. Unlike the case in Figure 17, a relatively large error is observed in the compressive signal’s time response against the original signal. It appears that it originates from the reconstructed signal’s RE against the original signal. As a way to overcome the compressive signal’s RE, a user can increase the number of filters against the RE, as described Figure 6, after measuring the efficiency in configuring the filter bank. In Figure 20, just like the characteristics of the reproduction of the reconstructed signal’s frequency response against the original signal shown in Figure 14, the compressive signals intensively simulate the mode information on the original signal’s frequency range (less than 10 Hz).

Compared to the original signal, the estimated compressive signal’s frequency response is 0.03598 in terms of the SE, showing about 63% of spectrum reconstruction ability against the total original signal’s frequency response. In particular, the SE on the originally targeted frequency range (less than 10 Hz) is 0.02831, showing about 71% of spectrum reconstruction ability. Based on the results, even though only several band-pass filters are used through the BOA, the AFB-added, the data compression technology-based IDAQ system developed in this study turns out to be effective in acquiring effective dynamic responses which can sufficiently express the targeted original signal’s time and frequency information by acquiring compressed peak values only through the PPA. In the restoration of compressive signals using peak values only, in addition, linear interpolation is effective in creating compressive signals within the complete peak value section while preserving conventional peak value information.

## 6. Conclusions

This study develops a data compression technology-based IDAQ system for the efficient by overcoming the limitations of the conventional hardware-based wired and wireless SHM system and adopting the latest measuring system and technology. To test the validity of the developed IDAQ system, the test is carried out. Then, the study results are as follows:

1. The BOA developed for the AFB highlights only the signals of the frequency range including the mode of interest among random signal’s broad frequency factors. The reconstructed signal reveals 70% and 83% of reconstruction effects in time and frequency responses against the original signal respectively. In particular, when the AFB is optimized using the El-Centro seismic waveform, it could be available as a filter technology needed to acquire dynamic responses in large flexible architectural structure (less than 10 Hz).
2. The central difference method-based PPA developed for the AFB selectively resamples only peak values including effective modal information among the reconstructed signals. Then, about 80% of data compression effects are expected. This kind of data compressing technology can overcome the limitations of RF communication and be available as a technology for efficient operation and management (big-data problem) of database at a long-term monitoring.
3. The AFB developed by using high-level language is embedded into the IDAQ system in a fast and accurate manner. As a result, it is able to acquire compressed effective dynamic responses on a real-time basis, specially, because the AFB can add or deduct diverse logics and functions depending on a user’s needs and easily edit and add the filter bank’s logic when response changes. Therefore, it can innovatively improve the conventional hardware-based filter bank’s design and implementation method in terms of efficiency and economy.
4. The linear interpolation applied for the restoration of the compressive signal is found to be effective in creating compressive signals for SHM by keeping the conventional reconstructed signal’s peak values intact. Then, in time and frequency responses against reconstructed signals, the compressive signal reveals 81% and 85% of expected reconstruction effects, respectively. This kind of linear interpolation can enhance the IDAQ system’s practical availability.
5. The IDAQ system overcomes the conventional hardware-based wired and wireless SHM system’s limitations by adopting high-performance hardware, multi-input/output channel (I/O), high-speed RF module, EST and RTOS based on the latest measuring system and technology. With the addition of AFB, in particular, it can substitute the conventional hardware-based filter bank, showing a possibility as a brand-new SHM system which enables efficient dynamic responses acquisition from structures.
6. In further studies, the IDAQ system would be continuously upgraded and applied to actual structures with a goal of evolving into a bio-inspired structural system equipped with intelligent sensing, judgment and response capabilities.

## Figures and Tables

**Table 1:** RE values for a filter bank with 10 filters.

		Bandwidth of Filters (Hz)																			
		0.1	0.2	0.3	0.4	0.5	0.6	0.7	0.8	0.9	1.0	1.1	1.2	1.3	1.4	1.5	1.6	1.7	1.8	1.9	2.0
Filter Spacing (Hz)	2.4	0.0351	0.0328	0.0309	0.0293	0.0280	0.0266	0.0253	0.0243	0.0234	0.0225	0.0217	0.0210	0.0203	0.0197	0.0192	0.0188	0.0185	0.0183	0.0181	0.0181
	2.2	0.0330	0.0306	0.0287	0.0272	0.0259	0.0247	0.0236	0.0226	0.0217	0.0207	0.0199	0.0192	0.0186	0.0180	0.0177	0.0175	0.0174	0.0174	0.0174	0.0075
	2.0	0.0335	0.0302	0.0276	0.0254	0.0235	0.0220	0.0206	0.0194	0.0183	0.0174	0.0167	0.0161	0.0157	0.0154	0.0153	0.0154	0.0157	0.0160	0.0163	0.0168
	1.8	0.0326	0.0295	0.0272	0.0252	0.0233	0.0216	0.0200	0.0186	0.0173	0.0162	0.0153	0.0147	0.0143	0.0141	0.0143	0.0146	0.0150	0.0157	0.0164	0.0173
	1.6	0.0329	0.0298	0.0272	0.0249	0.0228	0.0207	0.0186	0.0169	0.0155	0.0145	0.0140	0.0137	0.0137	0.0141	0.0148	0.0156	0.0165	0.0176	0.0187	0.0199
	1.4	0.0329	0.0296	0.0269	0.0244	0.0219	0.0194	0.0172	0.0155	0.0142	0.0135	0.0133	0.0136	0.0144	0.0154	0.0167	0.0181	0.0197	0.0214	0.0232	0.0251
	1.2	0.0316	0.0279	0.0246	0.0214	0.0184	0.0159	0.0141	0.0130	0.0126	0.0131	0.0141	0.0156	0.0174	0.0194	0.0217	0.0240	0.0264	0.0287	0.0311	0.0335
	1.0	0.0319	0.0271	0.0226	0.0184	0.0149	0.0127	0.0117	0.0121	0.0137	0.0160	0.0188	0.0218	0.0249	0.0281	0.0312	0.0343	0.0373	0.0403	0.0432	0.0459
	0.8	0.0308	0.0253	0.0201	0.0158	0.0128	0.0120	0.0139	0.0173	0.0212	0.0256	0.0300	0.0344	0.0386	0.0428	0.0469	0.0504	0.0538	0.0572	0.0605	0.0638
	0.6	0.0289	0.0221	0.0166	0.0138	0.0145	0.0187	0.0244	0.0306	0.0368	0.0429	0.0487	0.0537	0.0586	0.0633	0.0679	0.0724	0.0767	0.0809	0.0850	0.0890
	0.4	0.0262	0.0182	0.0150	0.0196	0.0274	0.0360	0.0446	0.0522	0.0596	0.0666	0.0734	0.0799	0.0860	0.0919	0.0976	0.1026	0.1075	0.1122	0.1168	0.1212
	0.2	0.022	0.0217	0.0323	0.0444	0.0567	0.0683	0.0791	0.0880	0.0960	0.1033	0.1099	0.1154	0.1204	0.1250	0.1292	0.1327	0.1359	0.1389	0.1417	0.1441

## Abbreviations

The following abbreviations are used in this manuscript:

SHM	Structural Health Monitoring
IDAQ	Intelligent Data Acquisition
EST	Embedded Software Technology
RTOS	Real-Time Operating System
DAQ	Data Acquisition
RF	Radio Frequency
AP	Access Point
AFB	Artificial Filter Bank
BOA	Band-pass filter Optimizing Algorithm
PPA	Peak-Picking Algorithm
RE	Reconstruction Error
CR	Compressive Ratio
SE	Spectrum Error
FFT	Fast Fourier Transform
